# Enhancing Dendritic Cell Cancer Vaccination: The Synergy of Immune Checkpoint Inhibitors in Combined Therapies

**DOI:** 10.3390/ijms25147509

**Published:** 2024-07-09

**Authors:** Serena Zanotta, Domenico Galati, Rosaria De Filippi, Antonio Pinto

**Affiliations:** 1 Hematology-Oncology and Stem-Cell Transplantation Unit, Department of Onco-Hematology and Innovative Diagnostics, Istituto Nazionale Tumori—IRCCS—Fondazione G. Pascale, 80131 Napoli, Italy; s.zanotta@istitutotumori.na.it (S.Z.); a.pinto@istitutotumori.na.it (A.P.); 2 Department of Clinical Medicine and Surgery, Università degli Studi di Napoli Federico II, 80131 Napoli, Italy; rdefilip@unina.it

**Keywords:** dendritic cells, dc-based cancer vaccine, immune checkpoint inhibitors, cancer therapy, combinatorial therapy

## Abstract

Dendritic cell (DC) cancer vaccines are a promising therapeutic approach, leveraging the immune system to fight tumors. These vaccines utilize DCs’ ability to present tumor-associated antigens to T cells, triggering a robust immune response. DC vaccine development has progressed through three generations. The first generation involved priming DCs with tumor-associated antigens or messenger RNA outside the body, showing limited clinical success. The second generation improved efficacy by using cytokine mixtures and specialized DC subsets to enhance immunogenicity. The third generation used blood-derived DCs to elicit a stronger immune response. Clinical trials indicate that cancer vaccines have lower toxicity than traditional cytotoxic treatments. However, achieving significant clinical responses with DC immunotherapy remains challenging. Combining DC vaccines with immune checkpoint inhibitors (ICIs), such as anticytotoxic T-lymphocyte Antigen 4 and antiprogrammed death-1 antibodies, has shown promise by enhancing T-cell responses and improving clinical outcomes. These combinations can transform non-inflamed tumors into inflamed ones, boosting ICIs’ efficacy. Current research is exploring new checkpoint targets like LAG-3, TIM-3, and TIGIT, considering their potential with DC vaccines. Additionally, engineering T cells with chimeric antigen receptors or T-cell receptors could further augment the antitumor response. This comprehensive strategy aims to enhance cancer immunotherapy, focusing on increased efficacy and improved patient survival rates.

## 1. Introduction

Cancer continues to be a significant global challenge, leading to over 9 million deaths in 2020 alone.

Traditional treatments, such as chemotherapy, radiation, and surgical interventions, have significant limitations and side effects, prompting the search for more innovative strategies. One promising approach involves cancer vaccination, using dendritic cells (DCs) to trigger the body’s immune response against tumors [1,2,3].

Dendritic cells, key players in the human immune system, display a rich diversity in their composition. This includes the conventional CD11C^+^ DCs (cDCs), with subsets such as cDC2 (CD1C^+^) and cDC1 (CD141^+^), along with the CD11C^−^ plasmacytoid DCs (pDCs) characterized by CD123^+^ and CD303^+^ cells. There’s a well-documented association between various diseases, including cancers and alterations in DC populations, either through a decrease in numbers or impaired functionality. Mature DCs are crucial for initiating the body’s immune defense against cancers by identifying and activating effector cells of the adaptive immune system. They are particularly adept at presenting tumor-associated antigens (TAAs) to T lymphocytes, thereby facilitating adaptive anticancer responses [4,5,6].

The tumor microenvironment can have a negative impact on DC maturation and function, leading to a state of tolerance that weakens the immune system’s ability to fight tumors. An efficient DC-based cancer vaccine must have both efficient TAA presentation and co-stimulating molecules to overcome the tumor’s immunosuppressive effects [4,5,6]. The ultimate objective is to harness DCs’ inherent capacity to penetrate tumors and elicit a robust immune response that involves T lymphocytes and natural killer (NK) cells [4,5,6,7]. Over recent years, DCs have emerged as a cornerstone of cancer immunotherapy, leading to the development of more effective clinical protocols and positioning DC-based vaccines at the forefront of this critical area of research [1,3,6].

## 2. DC-Based Cancer Immunotherapy

The last decade has witnessed significant efforts in refining DC vaccine technology. With more than 200 clinical trials targeting various cancers, including melanoma, prostate cancer, glioblastoma, and renal cell carcinoma, the advancements are noteworthy [8,9]. The development of DC vaccines can be categorized into three “generations”.

The first generation of DC vaccines was initially produced ex vivo from sources such as peripheral blood mononuclear cells (PBMCs), monocyte-derived DCs (MoDCs), or CD34^+^ hematopoietic progenitors [10,11].

These cells were then primed with tumor-associated antigens (TAAs) or messenger RNA (mRNA) encoding these antigens [11]. While this method was confirmed to be safe, its clinical efficacy was limited, with a reported tumor regression rate of only 3.3% [8,12]. This prompted a shift towards more sophisticated approaches in subsequent generations, aiming to enhance the efficacy of DC-based cancer vaccines by improving antigen presentation and the induction of a robust immune response against tumors.

The second-generation DC vaccines were developed to boost their immunogenicity by maturing and activating specific DC subsets using cytokine cocktails and pathogen-derived molecules [13,14].

This generation notably incorporated TAAs such as peptides from melanoma-associated antigens, Wilms tumor 1 (WT1), New York esophageal squamous cell carcinoma 1 (NY-ESO-1), and even whole tumor cell lysates [9]. The increased effectiveness of these DC vaccines is largely due to their utilization of more specialized DCs adept at presenting antigens through major histocompatibility complex (MHC)-I/II and initiating cytotoxic T lymphocyte (CTL) responses [15]. Specifically, pDCs are highlighted for their proficiency in Type I interferon (IFN) responses, while cDCs are recognized for their enhanced ability in engulfing dying cells and presenting antigens [16,17]. The strategic utilization of CD1c^+^ cDCs and CD141^+^ cDCs, both recognized for their high capacity for MHC-dependent antigen presentation, significantly enhances the proliferation of CTLs and the generation of CD8^+^ memory T cells, respectively [8].

Early clinical trials have shown objective responses in 5% to 15% of cancer patients receiving these vaccines, along with indications of improved survival rates [8]. However, a major challenge persists in consistently generating DCs that effectively target stable tumor antigens effectively and activate tumor-killing T cells across various patients.

This review will cover recent progress and challenges related to combinatorial strategies as promising anticancer treatments. Identifying immune checkpoint targets and combining DC-based immunotherapies with immune checkpoint inhibitors (ICIs) could have the potential to enhance the efficacy of DC-based immunotherapies for affected patients.

## 3. The Next Generation of DC-Based Vaccination

The field of DC-based vaccination is rapidly advancing, with a significant move towards using DCs directly sourced from the bloodstream. According to this approach, blood-derived DC subsets may cause a stronger immune response than those produced in vitro. An example of this is the intranodal injection of activated human pDCs combined with TAA peptides, which has been shown to induce significant T-cell responses in metastatic melanoma patients [18]. Clinical trials in prostate cancer and melanoma have not only confirmed the safety and feasibility of using blood-derived cDC2s CD1c^+^ with TAA peptides but also suggested an improvement in progression-free survival rates. Specifically, the use of autologous CD1c^+^ DCs has been linked to long-term progression-free survival (12–35 months) in a subset of melanoma patients [19,20]. Additionally, a study using autologous pDCs loaded with TAA peptides in melanoma patients reported antigen-specific CD4^+^/CD8^+^ T-cell responses and a measurable IFN signature [18].

Progress in DC vaccines also extends beyond the choice of cell type, with significant efforts focused on the precise selection of immunizing antigens. Attention is particularly focused on tumor-specific neoantigens, unique peptides resulting from somatic mutations absent in normal cells.

These neoantigens represent a promising target for enhancing the specificity and efficacy of DC vaccines [1]. In a phase I clinical trial focused on patients with advanced melanoma, the use of DC vaccinations, loaded with neoantigenic mutant peptides of high affinity and tailored to the individual profiles of the patients, has shown promising results. These vaccines have been successful in initiating neoantigen-specific T-cell responses, leading to disease stabilization or non-recurrence in some cases [21]. However, the variability and limited occurrence of neoantigens across different cancers and among patients present significant challenges. This variability necessitates the careful selection of inducers that are not only specific to the type of cancer but also, when possible, to the genetic makeup of the individual patient. Additionally, there are concerns about the potential for cross-reactivity with nonmutated proteins, which could trigger autoimmunity [22,23]. Nevertheless, more clinical insights are required to understand the potential efficacy of neoantigen-based DC vaccines.

Neoantigens can be introduced to DCs using various methods, such as pulsing with synthetic peptides, using autologous whole tumor lysate (WTL), or fusing with tumor cells, as well as pulsing with whole mRNA from autologous tumors.

Approaches utilizing the whole tumor cell have served as polyvalent sources of TAAs, capable of triggering a more comprehensive immune response [24,25]. This strategy aims to bypass the challenge of antigen selection by including a broad array of TAAs, including patient-specific neoantigens [24,25,26].

Early clinical trials have shown that, in order to effectively stimulate an antigen-specific immune response when encountering T cells, DCs need to be in a “mature” state [27].

Among the methods for introducing neoantigens into DCs, mRNA transfection stands out for its simplicity in generating intracellular neoantigens. Beyond just introducing neoantigens, mRNA electroporation can also incorporate functional proteins into the DCs, providing further activation and maturation signals [28]. Vaccines loaded with whole tumor mRNA have been shown to elicit specific T-cell responses against neoantigens and have been proven safe across a variety of tumors, including melanoma, renal, prostate, uterine and ovarian, colorectal, pancreatic cancers, multiple myeloma, and acute myeloid leukemia (AML) [29].

Cancer vaccines have demonstrated minimal toxicity in clinical trials compared to other cytotoxic therapies, encouraging the continued exploration of this immunotherapeutic strategy across various cancer types. However, achieving objective clinical responses with DC immunotherapy remains an elusive goal for specific cancer types [6,30,31,32].

## 4. Immune Checkpoint Inhibitors

Immune checkpoint inhibitors (ICIs) represent a novel class of immunotherapeutic agents that enhance the response of antitumor-specific T cells in a broad range of malignancies. The most commonly targeted molecules for their therapeutic potential include cytotoxic T-lymphocyte antigen (CTLA)-4, programmed death 1 (PD-1), and programmed death ligand 1 (PD-L1) [33,34,35]. Pembrolizumab and nivolumab are examples of anti-PD-1 monoclonal antibodies, while atezolizumab targets PD-L1, showcasing the breadth of strategies employed to bolster the immune system’s ability to fight cancer [36]. Immune checkpoint inhibitors have proven to be effective anticancer agents in the treatment of various cancers, including melanoma, lung cancer, bladder cancer, head and neck cancers, and Hodgkin and non-Hodgkin lymphoma [26,37,38,39,40]. Tumors with elevated mutational loads, such as melanoma and lung cancer, and those with a high frequency of mutational lesions, tend to respond better to ICI treatment [26,37,41]. This is because a higher tumor mutational burden leads to increased neoantigen load, which enhances the likelihood of generating immunogenic neoantigens. Notably, the activity of neoantigen-directed T cells in tumors that regress following ICI treatment has been a significant observation [42]. ICI treatment is particularly effective in tumors with an immune-inflamed phenotype, characterized by immune cell infiltration at the tumor edge or within the tumor stroma, indicating an inflammatory tumor environment. Conversely, patients with “non-inflamed” tumors and low neoantigen burdens may not respond as well to ICIs, underscoring the need for complementary strategies to enhance tumor-infiltrating lymphocytes [26,37,43,44]. In addition, the biology of specific tumors represents a critical aspect to be taken into account. As an example, tumor cells of Hodgkin lymphoma and of primary mediastinal B-cell lymphoma typically harbor structural alterations at chromosome 9p24.1, leading to the stable overexpression of PD-L1 and PD-L2 [45,46]. Consequently, the PD1-blockade has emerged as a major therapeutic strategy for both these malignancies [47,48].

The high clinical response rates achieved by ICIs have prompted the consideration of next-generation DC vaccines in oncology. DC-based cancer vaccines could play a crucial role in the success of ICI treatments by converting “non-inflamed” non-permissive tumors into “inflamed” tumors, thereby priming and recruiting intratumoral T cells.

## 5. ICIs in Combination with DC-Based Cancer Vaccination

DC vaccines ensure the antigen-specificity of the immune response, while a checkpoint blockade further amplifies it. Combining these approaches could enhance T-cell priming through the DC vaccine, especially when used with anti-CTLA-4 monoclonal antibodies, or prevent the exhaustion of vaccine-induced responses when paired with anti-PD-1 monoclonal antibodies. Preliminary in vitro data support the hypothesis of a synergistic effect between a checkpoint blockade and DC vaccination [49,50].

**Figure 1:**
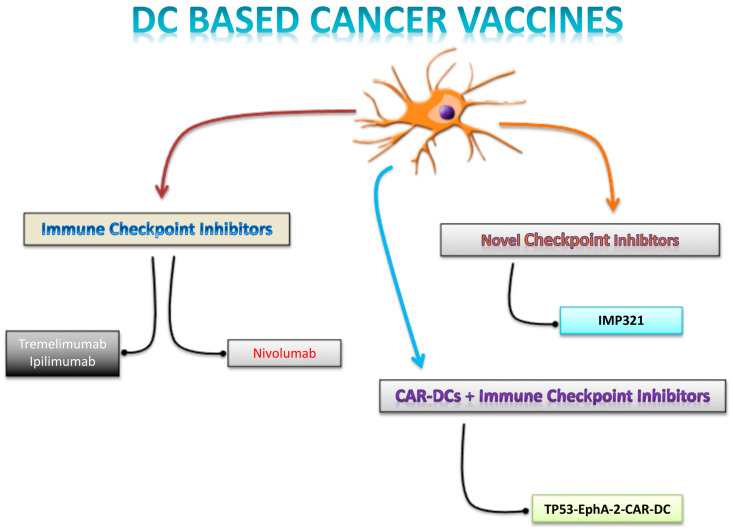
**Combinatorial strategies.** The Figure 1 systematically summarizes the use of immune checkpoint inhibitors based on the different therapeutic mechanisms, as combinatorial strategies to optimization of DC-based cancer vaccine.

Figure 1 summarizes the several approaches combining DC-based cancer vaccines with adjunctive strategies.

### 5.1. Anti-CTLA4 Antibodies

The data indicate that autologous DCs, pulsed with the melanoma antigen recognized by T cell 1 (MART-1) peptide, were administered alongside escalating doses of Tremelimumab (an anti-CTLA-4 antibody) to 16 patients with advanced melanoma. This treatment regimen was feasible and well-tolerated, with manageable dose-limiting toxicities. Antigen-specific T-cell responses were detected in 11 patients using MHC tetramer and enzyme-linked immuno SPOT (ELISPOT) assays. Additionally, gene expression profiling revealed that responders exhibited baseline overexpression of genes related to B-cell functions. In total, four patients showed objective tumor response, with two achieving partial response and two achieving complete response. These results suggest that combined immunotherapy offers a higher response rate compared to either single agent alone [33]. A subsequent phase II study in patients with pretreated advanced melanoma confirmed these promising results. This trial combined autologous DCs pulsed with melanoma-associated antigens (TriMixDC-MEL) and the CTLA-4-blocking antibody Ipilimumab. The combination significantly enhanced T-cell stimulation, as demonstrated by increased human leukocyte antigen (HLA)-DR expression and cytokine secretion in CD4^+^ T cells. The overall response rate was 38%, with eight patients achieving complete response and seven achieving partial response. The median follow-up for patients who responded was 36 months [51]. These findings indicate that Ipilimumab administration following the DC vaccine effectively boosts tumor-specific T-cell response [52]. Furthermore, the combination of the Mucin-1 (MUC1)-mRNA DC vaccine with CTLA-4 blockade outperformed either the DC vaccine or CTLA-4 blockade alone in patients with triple-negative breast cancer (TNBC) [52] (Figure 1).

### 5.2. Anti-PD-1 Antibodies

Monotherapy with anti-PD-1 is insufficient to suppress tumor growth, whereas combining anti-PD-1 treatment with a DC vaccine significantly suppresses melanoma growth in animal models [53]. Similarly, the human epidermal growth factor receptor 2 (HER2)-DC1 vaccine, when combined with anti-HER2 therapy and anti-PD-1, induces infiltration of both CD4 and CD8 T cells into tumors, leading to increased survival in preclinical models of HER2-positive breast cancer [54]. These findings align with reports that anti-PD-1 administration can enhance the efficacy of DC-based vaccines [55]. In this regard, data from multiple myeloma (MM) patients, as reported by Rosenblatt et al [56], show that PD-1 blockade enhances ex vivo T-cell response to DC vaccines in MM. Specifically, the anti-PD-1 antibody (CT-011) combined with autologous DCs fused with myeloma cells (DC/MM fusion) increases Th1 cytokine secretion, limits regulatory T-cell (Treg) expansion, and improves tumor cell killing [56] (Figure 1). Furthermore, a pilot study involving seven patients with stage IV pancreatic cancer found that low systemic doses of the anti-PD-1 antibody are more effective when paired with DC vaccination [57]. While clinical data is still pending, ongoing early-phase trials are actively investigating this synergistic approach with PD-1 blockade. One clinical study is assessing the effects of this combination strategy on MM patients compared to autologous transplantation alone. In this study, MM patients received serial infusions of the CT-011 antibody combined with the DC/myeloma fusion vaccine following autologous transplantation (NCT01067287).

Treatment with anti-PD-L1 can notably aid in the maturation of DCs and enhance the functionality of the DC1 subtype. This approach may also increase the number of activated cytotoxic T lymphocytes (CTLs) with strong antitumor properties. Consequently, combining DC vaccines with anti-PD-L1 could be a promising strategy for cancer therapy [58]. In a single-arm study (NCT02528682), researchers evaluated the effect of a PD-L1/L2-silenced, antigen-loaded DC vaccine in MM. The DC vaccine demonstrated minimal side effects, and no participants developed graft-versus-host disease (GVHD).

Genetic engineering is an option to deactivate ICI in the DC vaccine [59,60]. Currently, a clinical trial is examining the use of PD-L1/L2-silenced moDC in the post-transplant phase for various hematological malignancies (NCT02528682). This approach aims to prevent immune-related adverse events associated with checkpoint-inhibitor therapy [61] but may be less efficient as it will not prevent the inhibition of T cells by ligands expressed on leukemic cells [62].

Using patient-specific DCs combined with AML cells, a personalized cancer vaccine strategy showed promising results in stimulating antileukemic immune reactions, emphasizing the possibility of tailored vaccine treatments for AML patients once hematologic remission has been achieved [63]. This led to the initiation of a clinical trial to evaluate the effectiveness of AML DC hybrid vaccines, in combination with a blockade of the PDL-1/PD-1 pathway in a multicenter study performed in 63 AML subjects enrolled in April 2024 (NCT01096602).

A phase I/II trial (NCT03035331) is currently assessing the feasibility and efficacy of combining DC therapy with cryosurgery and Pembrolizumab in 11 non-Hodgkin lymphoma (NHL) patients enrolled as of April 2024. Cryosurgery works by freezing cancer cells, while immunotherapy involving monoclonal antibodies like pembrolizumab aims to enhance the body’s immune system in fighting the cancer and may interfere with the ability of tumor cells to grow and spread. A phase I/II trial is currently assessing the feasibility and efficacy of DC therapy in combination with cryosurgery and Pembrolizumab for patients with stage III-IV melanoma that is not surgically removable (NCT03325101).

Furthermore, in a phase II trial involving patients with follicular lymphoma (FL), a sequential intranodal immunotherapy (SIIT) approach is being utilized in conjunction with Pembrolizumab. This treatment method includes radiotherapy alongside ultrasound-guided injections of autologous DCs, rituximab, and granulocyte-macrophage colony-stimulating factor (GM-CSF) (NCT02677155).

Ongoing clinical trials are illustrated in Table 1.

## 6. Novel Checkpoint Targets

Recent research has been exploring novel checkpoint molecules for potential use in anticancer immunotherapy. These include T-cell immunoglobulin and mucin-containing protein-3 (TIM-3), lymphocyte activation gene-3 (LAG-3), and T-cell immunoglobulin and ITIM domain (TIGIT). They function as inhibitors of T-cell activation and are considered encouraging targets for therapy (Figure 1).

Blocking specific molecules alongside PD-1 on CD8 tumor-infiltrating lymphocytes (TILs) has been shown to be more effective than solely focusing on PD-1 in several studies. Based on preliminary research, blocking these pathways may enhance antitumor T-cell immune responses, possibly preventing tumor growth in solid tumors and hematologic neoplasms [64,65,66]. Advanced-stage melanoma patients were involved in a clinical trial where a combination therapy was administered. This therapy consisted of a MART-1 peptide-based vaccine and a LAG-3-Ig fusion protein (IMP321) targeting LAG-3’s natural ligand. IMP321 acts as a new adjuvant, triggering strong antigen-specific CD8 T-cell responses. While the trial did not show any objective clinical responses, it did reveal strong and long-lasting antitumor immune reactions in patients who were given IMP321. More specifically, there was a notable rise in tetramer-reactive MART-1 CD8^+^ T cells and a reduction in the levels of adaptive regulatory T cells (Tregs) [67]. The combination of these new checkpoint inhibitors with DC-based vaccines holds promise for improving immunogenicity and clinical efficacy, thus presenting hopeful possibilities for better clinical care of cancer patients.

Recent data show that blocking LAG-3 greatly enhances T-cell priming by DCs. This suggests that a potential promising treatment approach for malignancies with weak immune responses, like AML, could be the combination of a DC vaccine with anti-LAG-3 monoclonal antibodies [68]. Additionally, co-inhibition of PD-1, TIGIT, and TIM-3 following DC vaccination has been shown to stimulate CD8^+^ T-cell-mediated immune responses in gastric cancer models [69].

## 7. Enhancing the Efficacy of Combination Strategies with ICIs through Engineered T Cells

Autologous T cells engineered with T-cell receptors (TCRs) or chimeric antigen receptors (CARs) exhibit potent activation, specifically targeting and eliminating cancer cells. This innovative approach presents a compelling option for the treatment of various neoplasms. Clinical trials have validated the effectiveness of TCR- and CAR-engineered T cells in the treatment of diverse cancer types [70,71,72]. Expanding on these breakthroughs, a novel strategy could involve synergizing DC-based cancer vaccines with engineered T cells to amplify T-cell responses against tumor-associated antigens (TAAs). This combined approach holds promise in enhancing immune reactions against TAAs with limited immunogenicity (Figure 1).

In this regard, a pilot clinical trial has employed CAR-DCs containing an scFv domain targeting EphA2 antigen, loaded with TP53 mutant peptide (TP53-EphA-2-CAR-DC) in combination with ICIs to assess the safety and antitumor effects in subjects with local advanced/metastatic solid tumors or relapsed/refractory (R/R) lymphomas (NCT05631886).

Table 1 shows the ongoing clinical trials.

**Figure 2:**
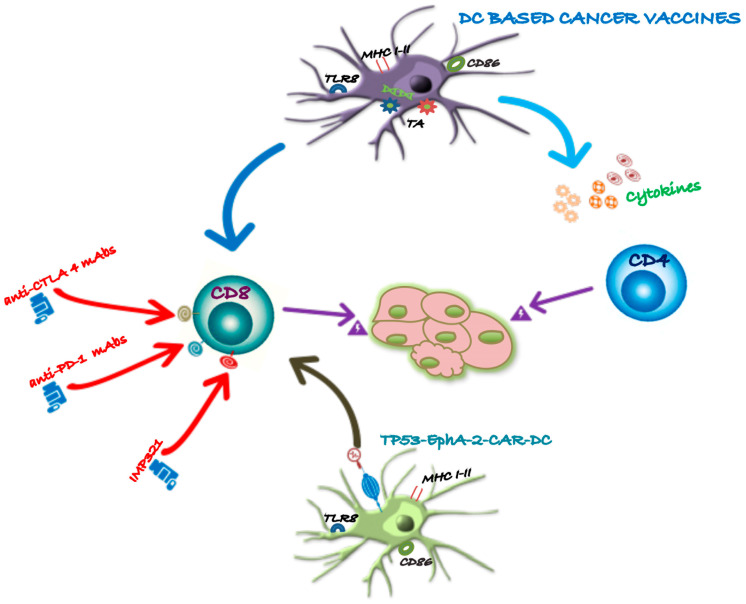
**Multimodal enhancement of DC-based cancer vaccines.** The Figure 2 demonstrates how combinatorial therapy with ICIs can enhance DC cancer vaccination. Checkpoint inhibitors are designed to augment the immune effector response and counteract immunosuppressive pathways. By synergizing DC-based cancer vaccines with engineered T cells, this approach can significantly amplify T-cell responses against TAAs, leading to a more effective and targeted antitumor immune response.

Figure 2 illustrates how checkpoint inhibitors promote antitumor-specific T-cell responses.

## 8. The Future of Cancer Immunotherapy

Recent clinical trials and research have validated the safety and feasibility of DC-based cancer vaccinations. However, the effectiveness of these vaccines in clinical settings is often hindered by the immunosuppressive nature of the tumor microenvironment (TME). The focus of current efforts is to unravel and reverse the complex interactions in the TME that disrupt immune function, impair T-cell activation, and prevent efficient cancer cell killing in tumors. Researchers are delving into various aspects of immune dysregulation to optimize T-cell function by employing advanced techniques for the generation, activation, maturation, in vivo targeting, antigen loading of DCs, and personalized mapping of neoantigens. The future of cancer immunotherapy is likely to revolve around the integration of these critical elements. This has led to an upsurge in clinical investigations that explore combining DC vaccinations with immunomodulatory agents, especially ICIs, to enhance therapeutic efficacy. Additionally, the potential synergy between DC-based cancer vaccination and cutting-edge immunotherapy approaches, such as CAR T-cell therapy, is being explored to amplify the antitumor immune response. The integration of multiple immunotherapeutic modalities has demonstrated improved efficacy and tolerability in clinical trials and sheds light on the complex landscape of cancer immunology. The identification of novel immune checkpoint molecules and the development of monoclonal antibodies against them [73] hold promise in enhancing the efficacy of DC-based cell therapy for cancer patients [74], although further clinical studies are warranted for translation into practice. Moreover, the convergence of next-generation sequencing (NGS) and bioinformatics tools is revolutionizing cancer immunotherapy by enabling the identification of tumor-specific neoantigens that can be utilized to design personalized DC vaccines [75,76]. Furthermore, advancements in nanotechnology are being harnessed to optimize the delivery and efficacy of DC vaccines. Engineered nanoparticles offer improved stability and bioavailability of antigens and adjuvants, facilitating targeted delivery to DCs and stimulating robust immune responses [77,78].

## 9. Conclusions

In conclusion, the future landscape of cancer immunotherapy is envisioned as a comprehensive approach that integrates DC-based vaccines with state-of-the-art technologies and novel therapeutic strategies. This holistic strategy aims to tackle the challenges posed by the TME and immune evasion mechanisms, ultimately enhancing patient outcomes and opening avenues for further advancements in the field.

## Figures and Tables

**Table 1:** **Active clinical trials with combinatorial strategies**. An organized summary of ongoing clinical trials on DC-based cancer vaccines with combination strategies.

Study ID	Therapeutic Strategy	Condition/Disease	Phase	Status
**NCT01067287**	CT-011 alone or plus DC fusion vaccine after ASCT	MM	II	Active, not recruiting
**NCT02528682**	MiHA-loaded PD-L-silenced DC Vaccination after allo-SCT	Hematological Malignancies	I/II	Completed
**NCT01096602**	Blockade of PD-1 plus DC/AML Vaccine after Chemotherapy	AML	II	Active, not recruiting
**NCT03035331**	Intratumorally DC vaccine After Cryoablation and Pembrolizumab	LNH	I/II	Active, not recruiting
**NCT03325101**	Intratumorally Autologous DC vaccine after Cryoablation plus Pembrolizumab	Melanoma	I/II	Active, not recruiting
**NCT02677155**	Sequential Intranodal Immunotherapy (SIIT) plus Pembrolizumab	FLC	II	Completed
**NCT05631886**	Intratumorally Autologous DC vaccine plus Cryoablation and Pembrolizumab	LNH	I/II	Active, not recruiting

**AML:** acute myeloid leukemia; **MM:** multiple myeloma.

## Data Availability

Not applicable.
